# Impact of probe tilt on Graf ultrasonography accuracy for neonatal hip dysplasia screening

**DOI:** 10.1051/sicotj/2025016

**Published:** 2025-04-01

**Authors:** Spyridon Sioutis, Stylianos Kolovos, Maria-Eleni Papakonstantinou, Pavlos Altsitzioglou, Maria Polyzou, Konstantinos Chlapoutakis, Vasileios Karampikas, Panayiotis Gavriil, Evanthia Mitsiokapa, Dimitrios Koulalis, Panayiotis J Papagelopoulos, Andreas F Mavrogenis

**Affiliations:** 1 First Department of Orthopaedics, National and Kapodistrian University of Athens, University Medical School 12462 Chaidari Athens Greece; 2 Department of Orthopaedics, General Hospital of Larisa 41221 Larisa Greece; 3 Third Department of Paediatrics, National and Kapodistrian University of Athens 12462 Athens Greece; 4 Rheumazentrum Ruhrgebiet, Ruhr-University Bochum 44649 Herne Germany; 5 Vioapeikonisi – Diagnostic Imaging Laboratory 71202 Heraklion Crete Greece; 6 Department of Physical and Rehabilitation Medicine, Thoracic Diseases General Hospital Sotiria 11527 Athens Greece

**Keywords:** DDH, Graf method, Hip Ultrasonography, Tilt Effect

## Abstract

*Background/objective*: Developmental Dysplasia of the Hip (DDH) is the most common congenital musculoskeletal disease of the infantile age. The gold standard for early diagnosis of the disease is the Graf ultrasound method. In our study, we examined the correlation between age of the examined infant and diagnostic errors due to the ultrasound probe tilt effect during examination. *Methods*: Forty-two newborns who underwent ultrasound examination with the Graf method, were included. We categorized the neonates into three age groups (Group#1: 0–1 weeks, Group#2: 3–4 weeks, Group#3: 5–6weeks). Two ultrasound examinations were performed in every group. In the first examination, images were obtained with the probe in vertical position. In the second examination, images were taken with a 10° caudocranial tilt of the probe. Our aim was to measure the *α* angle in both examination and to mention the possible Type changes according to the Graf classification. The *α* angle defines the osseous coverage of the femoral head from the acetabulum in the neonatal hip joint. *Results*: In many cases, the classification changed from type I to type IIa or D and from type IIa to D, when instead of the vertical acquisition, the ultrasound probe was placed in a 10° caudocranial tilt at the hip joint of the examined infant. At Group#1 of the study we observed 60 Graf classification Type changes (90.91%), while in Group#2 and Group#3 we had 18 (33.33%) and 3 (7.96%) Type changes respectively. *Conclusion*: As the age of the examined newborns increases, measurement and classification errors due to the tilt effect are significantly reduced. Clinically, the examination will be even more accurate and the use of an incorrect therapeutic approach due to incorrect classification will be avoided. Finally, the optimal time for conducting an ultrasonographic examination is between the 5th and 6th week of life.

## Introduction

The Graf method is a widely used ultrasonographic technique for the early diagnosis of the Developmental Dysplasia of the Hip (DDH) [1]. This method is based on the identification of the neonate hip joint’s structures and detects various types of dysplasia through measuring certain angles (*α* and *β*) that represent the osseous and the cartilaginous coverage of the femoral head by the acetabulum, respectively. During the examination, the ultrasonographic probe should be in strict vertical position to the hip joint. For this reason a specialized device (SonoFix – SonoGuide) should be used [2]. The physician should be familiar with the Graf method and precisely follow the rules of anatomy during the ultrasound examination.

In this study, we examined if the neonate’s age is related to diagnostic errors caused by the tilt of the ultrasonographic probe. Diagnostic errors during ultrasonography lead to errors in Graf classification. Every Type in the Graf classification leads to different therapeutic approach [3]. Consequently, there may be errors in the management of the neonates. Such mistakes can turn out to be critical, as the treatment of DDH is a sensitive process. For example, if Type I, healthy, neonates are classified as Type IIa, they will be unnecessarily treated as pathological cases. Additionally, a misclassification in pathological cases would lead to inadequate treatment and problems during the child’s developmental process. Our aim was to define the optimal period for the ultrasonographic examination, in which the measurement of the *α* angle and the type of the joint, do not change in spite of an extreme tilt effect.

We performed two different ultrasonographic evaluations using the Graf method. The first evaluation was carried out with vertical direction of the probe to the hip joint, while the second evaluation was carried out with a caudocranial tilt of 10°. We performed the ultrasonographic examination in three different periods (Group#1: 0–1 weeks, Group#2: 3–4 weeks and Group#3: 5–6 weeks) in order to assess how the tilt effect is affected by the age of the examined neonates. The 10° tilt was established after several attempts to find a golden value, where the correct (vertical position of the probe) from the false (tilt effect) ultrasonographic images obtained, could not be easily distinguished, while simultaneously the classification errors were significant. Tilting errors were observed when our examinations reached the extreme borders of false techniques. In reality, this is almost impossible, when the examiner uses the SonoFix – Sonoguide system. We also aimed to evaluate the limits of the Graf method and to find a safe zone.

## Materials and Methods

We conducted screening ultrasonographic examination with the Graf method, performing two different evaluations for each hip. During the initial examination the probe was put in vertical position to the hip joint, while in the second examination we performed the ultrasonography with the probe in a caudocranial tilt of 10°. The 10° tilt was selected after many tests to find the point where there is difference in the measurements, while at the same time respecting the anatomy and usability rules of the Graf method [4]. The researchers were experienced clinicians, dealing with pediatric orthopedics and trained in certified courses on the use of the Graf method.

We included 42 newborns (84 hip joints), who underwent ultrasound examination of both hip joints with the Graf method. We used the A8 sonoscape (7–12 MHz) and the SonoFix – Sonoguide system (Gebr. HIRSCHBECK GmbH ©, Austria) [2]. Finally, 252 accurate ultrasonographic images of the examined hip joints (84 hip joints, examination in 3 groups) and 252 ultrasonographic images that where obtained, while applying the 10° caudocranial tilt (84 hip joints, examination in 3 groups). All the ultrasonographic images were evaluated according to anatomical and usability rules of the Graf method. The pairs of images that did not meet the criteria were excluded [5]. More specifically, 18 pairs of images in Group#1, 30 pairs of images in Group#2 and 19 pairs of images in Group#3 were excluded. The number of the final pairs of images was 66 for the Group#1, 54 for the Group#2 and 63 for the Group#3. Initially taken pictures, with the probe in vertical position, were used to characterize the patients according to the Graf classification (Table 1). Thereafter, we classified the patient, by assessing the images obtained with the probe in 10° caudocranial tilt (Table 2).

**Table 1:** Graf classification using correctly the ultrasonographic probe (vertical position to the hip joint).

	Type I	Type D	Type IIa	Total
Group#1	53/66 (80.30%)	0/66 (0%)	13/66 (19.70%)	66/66 (100%)
Group#2	52/54 (96.30%)	0/54 (0%)	2/54 (3.70%)	54/54 (100%)
Group#3	63/63 (100%)	0/63 (0%)	0/63 (0%)	63/63 (100%)

**Table 2:** Graf classification using of the ultrasonographic probe in false position (10° craniocaudal tilt).

	Type I	Type D	Type IIa	Total
Group#1	0/66 (0%)	10/66 (15.15%)	56/66 (84.85%)	66/66 (100%)
Group#2	34/54 (62.96%)	0/54 (0%)	20/54 (37.04%)	54/54 (100%)
Group#3	58/63 (92.06%)	0/63 (0%)	5/63 (7.94%)	63/63 (100%)

The measurement of *α* angle was used to classify the hip joints according to the Graf classification [3]. We refrained from including the measurements of the *β* angle for statistical analysis, taking into consideration that they do not differentiate the categorization of our cases. The ultrasound examination at Group#1 was carried out as necessary screening test for DDH. In the other groups (#2 and #3) we monitored the progress and the changes in the examined hip joints.

Statistical analysis was conducted using IBM SPSS Statistics for Windows (Version 20.0; IBM Corp., 2011). The analysis builds on statistical inference techniques and on the construction of 95% confidence-intervals (CIs) for population proportions [6, 7]. In general, a (1–*a*)% CI of a population’s parameter *θ* (theta) expresses the interval where parameter *θ* lies under probability (1–*a*) whether submitted to independent samplings, as expressed by the relation:

(1) $$P\left\{\mathrm{lb}(X)\leq \theta \leq \mathrm{ub}(X)\right\}=1-a,$$

where *lb*(*X*) and *ub*(*X*) are random variables (associated to variable *X*) expressing the lower and upper bound of the CI, *θ* is the statistical parameter, and *a* is the so-called level of significance. As far as the population proportion *θ* = *p* is concerned, a (1–*a*)% CI is estimated by the mathematical formula:

(2) $$p_{\mathrm{lb}},p_{\mathrm{ub}}=p\pm z_{a/2},\;\sqrt{\frac{p\left(1-p\right)}{n}},$$

where *z*_*a*/2_ is the *z*-score for and *a*% level of significance, and *n* is the sample size. For the purpose of this analysis, we use in our computations by default 95% confidence intervals (*a* = 5%) [8, 9]. The cross-tabulation of the frequency percentages between the real and false type (intentionally submitted to tilt) measurements of the examined infants, per sampling period (Group#1: 0–1 weeks, Group#2: 3–4 weeks and Group#3: 5–6 weeks) are also demonstrated. Percentages are further analyzed in 95% confidence intervals, to statistically evaluate differences between transition states.

## Results

For the first sampling period (Group#1: 0–1 weeks), tilt applied to Type I neonates induced on average 5.66% (range 0–11.88%, under 95% certainty) shifts to Type D (I→D) and 94.34% (88.12%–100%) to Type IIa (I→IIa). Furthermore, tilts applied to Type IIa newborns induced on average 53.85% (26.75–80.95%) shifts to type D (IIa→D), whereas 46.15% (19.05%–73.25%) of these cases remained Type IIa (maintaining the same class, IIa→IIa).

For the second sampling period (Group#2: 3–4 weeks), tilts applied to Type I newborns on average 65.38% (52.45–78.32%) do not cause any shift (I→I), whereas they cause on average 34.62% (21.68%–47.55%) a shift to Type IIa (I→IIa). Moreover, tilts applied to Type IIa newborns do not cause any shifts to other Type (IIa→IIa).

Finally, for the third sampling period (5–6 weeks), only tilts applied to type I infants caused one Type change (I→IIa) on average 7.94% (0.66–14.61%), whereas 92.06% (84.79%–98.74%) newborns remained in type I after the 10° tilt application (I→I).

By comparing the correlation coefficient intervals across the sampling periods, we noted the following observations. For the shift “I→IIa”, we can conclude that shifts due to tilt effect statistically reduce over time (94.34%, statistical range between 88.12% and 100%) 0–1 week > (34.62%, statistical range between 21.68% and 47.55%) 3–4 weeks > (7.94%, statistical range between 0.66 and 14.61%) 5–6 weeks. Similarly, the shifts “I→D IIa→D” due to tilt effect observed only in Group#1 (0–1 weeks). In Group#2 and in Group#3 the hip joints that were classified as Type I in the correct measurement, either remained in Type I, or changed to Type IIa in the false measurement (10° caudocranial tilt). Accordingly, images classified as Type IIa in the correct measurement remain unchanged.

Consequently, the overall Type changes caused by the 10° caudocranial tilt tend to decrease as the age of newborns increase. To be more precise, at Group#1 we have 60 Type changes (Type I to Type IIa and Type IIa to Type D) among the 66 examined hip joints, a percentage of 90.91%. In Group#2 we observe 18 Type changes (Type I to Type IIa) among the 54 examined hip joints, a percentage of 33.33% and in Group#3 we only have 5 Type changes among the 63 examined hip joints, a percentage of 7.94% (Table 3).

**Table 3:** Frequency of absolute and percentage values of the Graf classification Type shifts between correct and false measurements, per sampling period (Group#1, Group#2 & Group#3).

Type shifts	Group#1	Group#2	Group#3
Type I to Type I	0/53 (0%)	34/52 (65.38%)	58/63 (92.06%)
Type I to Type IIa	50/53 (94.34%)	18/52 (34.62%)	5/63 (7.94%)
Type I to Type D	3/53 (5.66%)	0/52 (0%)	0/63 (0%)
Type IIa to Type IIa	6/13 (46.15%)	2/2 (100%)	–
Type IIa to Type D	7/13 (53.85%)	0/2 (0%)	–
Overall shifts	60/66 (90.91%)	18/54 (33.33%)	5/63 (7.94%)

* 95% CIs for the percentage*.

The *α* angle represents the coverage of the femoral head by the bony part of the acetabulum. In particular, neonatal hips with angle *α* angle values greater than 60 degrees are classified as Type I. *α* angles values of 50–59° are classified as Type IIa and angle *α* angle values between 43 and 49 degrees are classified as Type D. The importance of the Graf classification lies in the therapeutic approach. Hips classified as Type I are considered normal and do not need any therapeutic intervention. Type IIa hips require repeat ultrasound monitoring. Finally, type D hips require a therapeutic approach using a plaster. Consequently, errors in the measurement of angle *α* due to tilt affect the classification of the examined hip and may lead to significant errors in treatment.

**Figure 1:**
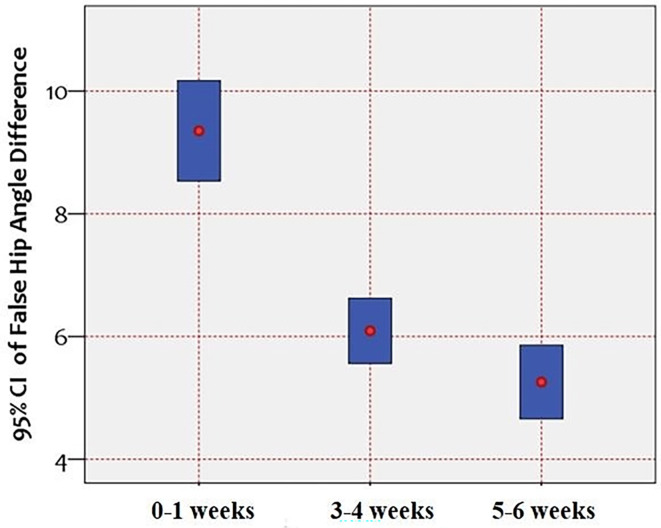
Error bars of 95% confidence intervals showing the *α*-angle difference (vertical axis, measured in degrees) between the vertical ultrasonographic examination and the examination with 10° caudocranial tilt of the sonographic probe, across the three groups of the study referring to sampling periods.

The *α* angle difference between the vertical and the 10° caudocranial tilt showed a remarkable reduction over time. More specifically, we observe a reduction from 9.35° in the Group#1 (0–1 weeks), to 6.10° in the Group#2 (3–4 weeks) and finally to 5.25° in the Group#3 (5–6 weeks). This succession illustrates a declining trend that shows that over time the 10^ο^ caudocranial tilt affects significantly less the correct measurement of *α* angle. The errors in Graf classification are minimized, increasing the safety and validity of the method (Figure 1).

Our analysis showed that unintentional errors due to tilt, statistically decrease through time. More accurate measurements may be potentially obtained in the period between 3–6 weeks (Group#2 and Group#3) and especially between the 5th and 6th week (Group#3).

It should be noticed that we properly completed the follow-up for the 13 cases that were correctly classified as Type IIa at Group#1 and the 2 of them that remained Type IIa at Group#2. They were finally classified as Type I at Group#3.

## Discussion

Developmental Dysplasia of the Hip (DDH) is the most common musculoskeletal disease during infantile age. Female gender, left hip, firstborn child, breech position in utero life, positive family history, oligohydramnios and specific syndromes consist of the major risk factors [10–16]. The pathophysiology of the disease is based on the abnormal development of the joint. DDH can range from dysplasia to dislocation of the femoral head from the acetabulum.

Early diagnosis of DDH is crucial as conservative means could be implemented for its treatment, instead of complex surgical operations [17]. The first stage of the diagnostic process is the clinical examination (Barlow and Ortolani maneuvers) [18]. The ultrasonographic examination has been considered as the gold standard for a definitive diagnosis of DDH. Graf method is the most popular among all ultrasonographic methods, enabling the physician to make a definitive diagnosis; to classify the examined infantile hip joint according to the Graf classification and to immediately apply the appropriate conservative treatment [4, 19, 20].

Although the Graf method is considered as a simple process, there are certain steps for an accurate diagnosis. It is necessary to use the Sonofix – Sonoguide equipment, which stabilizes the infant in the ideal lateral position. During the procedure it is important to adhere to the following rules, which are reported as check list 1 (anatomical identification) and check list 2 (usability check). The check list 1 includes the recognition of the following anatomical structures: 1. chondroosseous border, 2. femoral head, 3. synovial fold, 4. joint capsule, 5. lambrum, 6. hyaline cartilaginous roof, 7. bony roof and 8. bony rim (turning point from concavity to convexity). The check list 2 includes the recognition of the lower limb, the correct plane and the labrum in order to ensure that the probe is aiming to the center of the acetabulum [3, 10]. These two check lists guarantee also the verticality of the sonographic probe throughout the process and the detection of any potential significant tilt or other possible confounds.

The most common confounders of the Graf method derive from inter-operator variability, neonatal movement during scans and from the probe tilt effect. The tilt is caused by the non-vertical positioning of the sonographic probe in relation to the hip joint [21]. Performing the ultrasonographic examination with an average 10° caudocranial tilt of the probe, we examined how the measurement of angle *α* changed and therefore if there were any shifts in the Graf classification and if the incidence of the shifts changed through time.

**Figure 2:**
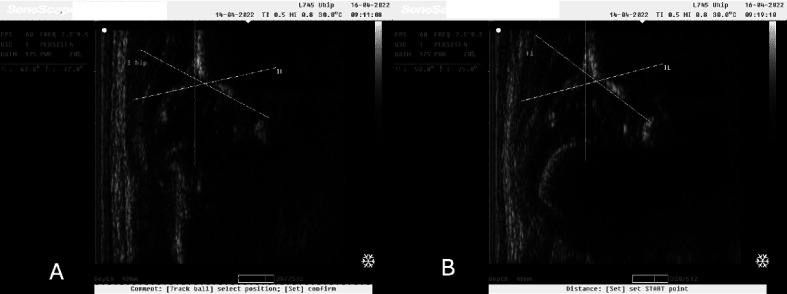
A: Type I hip joint (*α* angle 62°) at Group#1 (vertical view of the sonographic probe the hip joint), B: Type IIa hip joint (*α* angle 53°) at Group#1 (10 ocaudocranial tilt view of the probe to the hip joint). This image illustrates the same hip joint, in the same time of examination.

**Figure 3:**
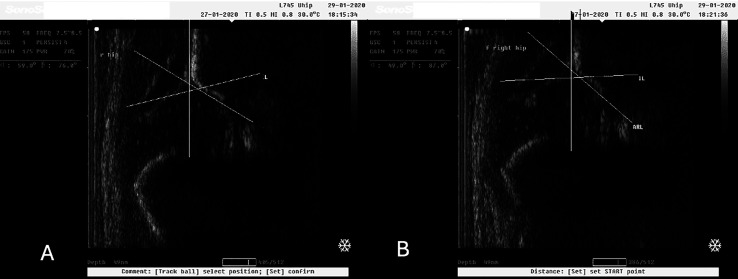
A: Type IIa hip joint (*α* angle 59°) at Group#1 (vertical view of the (Sonographic) probe to the hip joint), B: Type D hip joint (*α* angle 49°) at Group#1 (10° caudocranial tilt view of the probe to the hip joint). This image illustrates the same hip joint, in the same time of examination.

**Figure 4:**
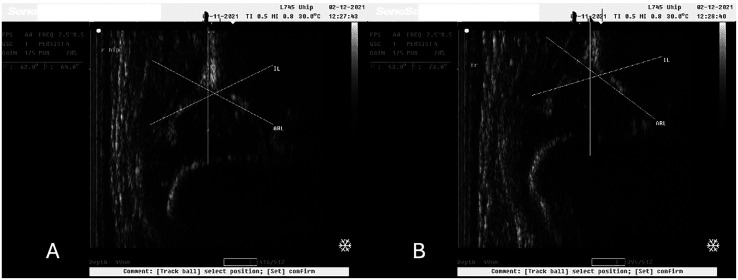
A: Type I hip joint (*α* angle 62°) at Group#2 (vertical view of the probe to the hip joint), B: Type IIa hip joint (*α* angle 53°) at Group#2 (10° caudocranial tilt view of the probe to the hip joint). This image illustrates the same hip joint, at the same time of examination.

**Figure 5:**
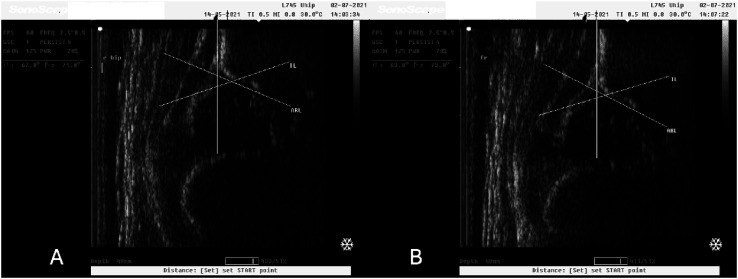
A: Type I hip joint (*α* angle 67°) at Group#3 (vertical view of the sonographic probe to the hip joint), B: Type I hip joint (*α* angle 63°) at Group#3 (10° caudocranial tilt view of the probe to the hip joint). This image illustrates the same hip joint, at the same time of examination.

In our study we observed normal hip joints (Type I) that changed to Type IIa after the application of 10° caudocranial tilt (Figures 2a and 2b). Still, there were Type IIa hip joints that also changed to Type D after the applied tilt on the ultrasonographic probe (Figures 3a and 3b). These images were captured during ultrasonographic examination at the Group#1 of the study. Additionally, there were also normal hip joints (Type I) that changed to Type IIa after the application of 10° caudocranial tilt at the Group#2 of the study (Figures 4a and 4b). Normal hip joint (Type I) remained Type I after the application of 10° caudocranial tilt at the Group#3 of the study (Figures 5a and 5b).

According to our results, the Graf method becomes more reliable between the 5th and 6th. As the age increases (Group#2 and Group#3), the tilt effect does not affect significantly the ultrasonographic measurement of the *α* angle and rarely changes the Graf classification type of the infant hip joint. Thus, there is no confusion in the physician’s decision as to whether a therapeutic approach should be applied and which it would be.

According to other studies, most errors in the Graf method result from the incorrect identification of the anatomical structures of the examined hip joint. Significant errors also arise from the use of sector or trapezoid probes. Due to the structure of the joint with bone, cartilage and soft tissue only linear probes should be mentioned. In the these cases there is a tendency for overdiagnosis DDH [22, 23]. In other studies, faulty training of the examiners is mentioned as a major factor of errors in the Graf method. Specifically, it was observed that examiners who have not been trained in a specialized seminar tend to make more wrong measurements. In these cases, more hips are usually recorded as type I, when in fact they should be classified as type IIA, or IIb [24, 25]. Other reasons for mistakes are the wrong placement of the examined neonate and the free-hand technique. When the rules of anatomy and usability are strictly followed by an experienced examiner, the Graf method is very accurate and useful for the treatment of DDH.

There is limitation on the timeline that the ultrasonographic evaluation can be carried out for technical reasons. Additionally, late diagnosis, after 3 months of life, possibly leads to inadequate treatment and to complications. Respectively, very early examination may lead to false-positive results [26–28]. The increased reliability of the Graf method between the 5th to 6th week of life, and the ability to start the therapeutic intervention within this period is a serious argument to suggest it as the optimal time frame to perform the ultrasonography examination.

The clinical significance of errors due to the tilt effect is very important. A wrong measurement in *α* angle may cause a Type shift in Graf classification and use of an inappropriate treatment protocol. DDH is a severe condition. The diagnosis needs accuracy and certainty, because treatment failure will affect hip joint’s growth and may cause stability and kinesiology problems during adolescence and in adult life for the patient.

Limitations of the study were that there were no neonates with Graf type III or IV hip joints and that neonates with potential co-morbidities, presence of any syndromes or preterm birth were excluded. Further research is welcome for the optimization of the Graf method. An area with great interest for future research would be the study of the ratio of the femoral head dimensions on ultrasound examination as a predictor of validity.

Summarizing, to minimize misclassification due to the probe tilt, neonatal hip ultrasonography should ideally be performed between the 5th and 6th weeks of life. Even if there is an error due to the tilt effect, it does not affect significantly the Graf classification and the selection of the proper treatment approach.

## Data Availability

The data are available from the corresponding author upon reasonable request.
